# Conservative and cost‐effective rehabilitation of a deep bite patient with worn dentition: A case report of the Dahl technique approach

**DOI:** 10.1002/ccr3.8058

**Published:** 2023-10-17

**Authors:** Mahla Rezaei, Hossein Chalakinia, Maedeh Doost Mohammadi, Farzaneh Khosravi

**Affiliations:** ^1^ Department of Restorative and Cosmetic Dentistry School University of Dentistry Mashhad University of Medical Sciences Mashhad Iran; ^2^ Mashhad University of medical sciences Mashhad Iran

**Keywords:** Dahl technique, non‐carious cervical lesion, rehabilitation, tooth wear, vertical dimension loss

## Abstract

The reformation of teeth with severe wear is a real challenge for clinicians. Through a clinical case study, this case illustrates a minimally invasive approach using the Dahl technique and bonded restorations for the treatment of a worn dentition with a loss of vertical dimension of occlusion and restoring ideal aesthetics.

## 1 INTRODUCTION

The general term for non‐carious loss of dental tissue non‐carious cervical lesion (NCCL) is tooth wear which describes the loss of hard dental tissue due to various etiological factors, including chemical and mechanical processes.^1^ According to Loomans et al., tooth wear, when abnormal for the age of the patient, causes pain and discomfort, functional issues, or decline in aesthetic appearance, and if progressing, may result in complex undesirable complications. Tooth wear can be classified as erosion, abrasion, attrition, and abstraction.^2^ The clinical observations have shown that the wear mechanisms do not act alone but interact with each other and usually with synergic effect on each other to destroy dental surfaces.^3^ The increased occurrence of erosive wear has been reported in young populations due to dietary habits, a growing tendency to consume soft and energy drinks, and the impact of stress. Regardless of the causative factors, tooth wear may affect oral health, personal comfort, loss of vertical dimension of occlusion (VDO), tooth sensitivity, hyperactive muscles, temporomandibular joint problems, and pulpal involvement.^4^ However, aesthetic complaints exist, particularly in young adult patients. The incisal edges may also show signs of wear, shortening due to the loss of enamel support, and subsequent fracture, depending on the progression. A high prevalence of tooth wear has been reported in the young population, which can be reflective of future dental problems in this generation. Also, in the elderly population, complete loss of dentition is now second to severe tooth wear as a dental problem. For successful treatment of the above cases, paying close attention to the anterior guidance, posterior contacts, and TMJ is essential.^5^ The key benefit of a dental filling is halting the progression of decay by repairing a damaged tooth and restoring its normal function and appearance. Fillings are used to fix small areas of decay and they prevent further deterioration by providing a barrier against harmful bacteria.

### 1.1 The significance of anterior guidance

Following centric relation (CR), anterior guidance is the most significant factor that must be determined when restoring an occlusion. Aside from its key role in aesthetics, anterior guidance is a crucial factor in protecting the posterior teeth. This protective role of anterior guidance is so important that if the posterior teeth are not protected from lateral and protrusive stresses by the separating effect of the anterior teeth, they will be subjected to high and non‐vertical stress over time which can lead to the worn dentition. Patient comfort is determined by how accurately the anterior guidance is coordinated with the functional pattern of other parts of the masticatory system.^6^

### 1.2 The consent statement

She read the information and has had an opportunity to ask questions about the research and how her information was used. She understood the purpose of the study and what her participation involved. She agreed to participate in conservative and cost‐effective rehabilitation of a deep bite with worn dentition and for the information. She understood she needed to inform the other members of my family that she has given consent which allowed them to opt‐out if they wish.

She understood that anonymized information about her and her family may be published within the project report, which may be published online, and that published material from this report may be used and distributed for training, service design, and development.

She knew that her participation was voluntary and that she could choose to withdraw from the research at any point.

### 1.3 Posterior–anterior contacts

The following formula should be kept in mind when analyzing the anterior guidance: “*Lines in the front and dots in the back*.” The dots in the back merely show the contact points on posterior teeth. The lines in the front demonstrate the role of anterior teeth in separating the posterior teeth during all eccentric movements. Thus, the stable contacts of anterior teeth in CR and sliding contacts in lateral movements are fundamental issues in extensive restorations.^7^ This is why this scheme is called a “mutually protected system”.

### 1.4 Treatment options

The biggest problem with restoring worn dentition is there is no space for restorative material to provide optimum resistance and retention form. A variety of treatment options have been proposed, including elective endodontic treatment, surgical crown lengthening (CL) for restoration of worn teeth with insufficient restorative space, and prosthetic treatments.^8^ However, these conventional methods are very costly, time‐consuming, and invasive. They destroy a considerable amount of tooth structure and are accounted as an irreversible path for both the patient and dentist. Another problem with these invasive treatment plans is they are heavily dependent on laboratory precision, in fact, if the dentist has no access to a skillful laboratory whole treatment can lead to a failure. With advances in adhesion and dental bonding agents, minimally invasive restorations have been introduced to preserve the residual tooth structure. More invasive approaches as the treatment options can be postponed until more advanced ages.

An alternative treatment option is occlusal veneers which are considered a conservative approach to increasing the VDO in cases with severely worn teeth. The durability of these restorations and their ease of construction make them an appropriate conservative treatment option.^9^ But they are costly and still need a skillful ceramist.

An ultra‐conservative and simple treatment is proposed using a combination of composite resin and the Dahl principle to resolve the anterior teeth wear. The Dahl approach involves the creation of inter‐occlusal space through axial movement of the teeth via an appliance or the restorations placed in the supra‐occlusion and subsequently re‐creating the occlusal contacts of the full arch over a while. The primary Dahl appliance is based on a metal cobalt‐chromium appliance cemented on the palatal surfaces of the upper anterior teeth.^10^ However, the quality and long‐term esthetical properties of directly bonded restorations are more dependent on the operator, they have been used recently to create the proper space with low to moderate durability compared to indirect restorations.^11^ Concerning the directly bonded restorations, as stated by Craig, while using the composites for the restoration of worn teeth is superior to using ceramics due to their lower modulus of elasticity and seemingly satisfactory clinical performance, they require maintenance owing to their limited mechanical and physical properties.^12^

## 2 CASE REPORT

**FIGURE 1:**
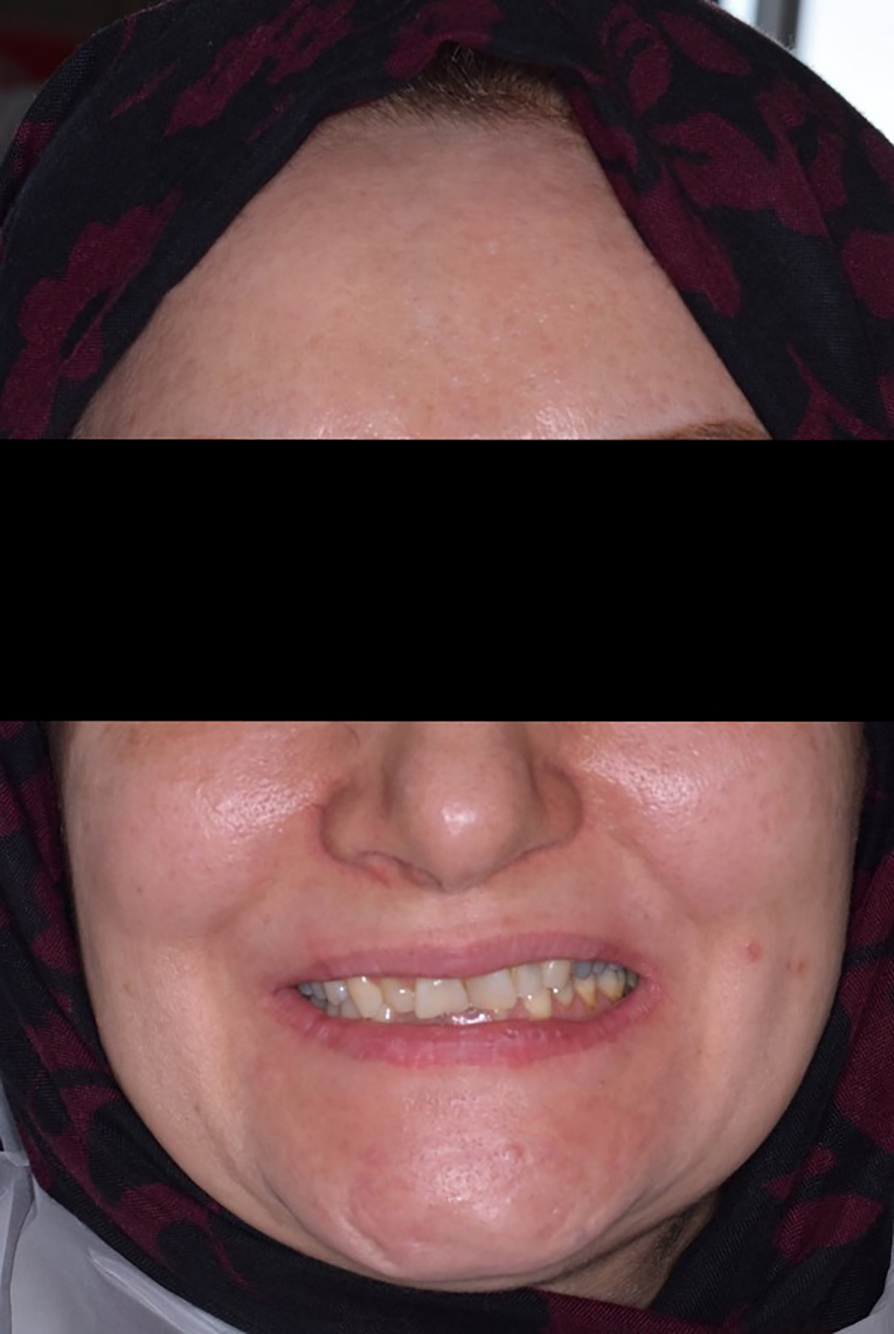
Frontal smile view of the patient.

The patient was a 36‐year‐old woman without any systemic disease with the chief complaint of unaesthetic teeth and an unpleasant feeling of tooth grinding during sleep (Figure 1).

**FIGURE 2:**
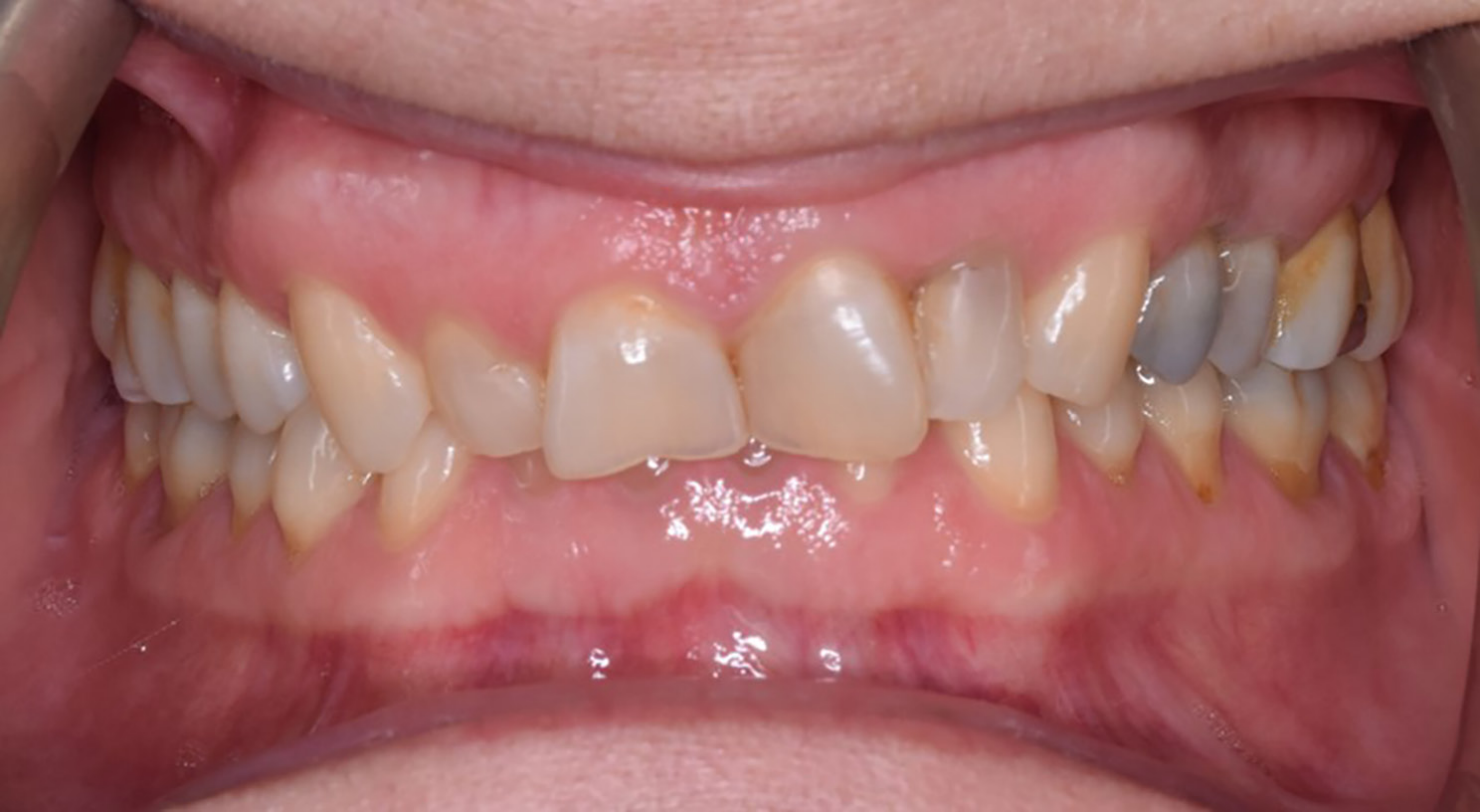
The intraoral frontal view of the patient.

**FIGURE 3:**
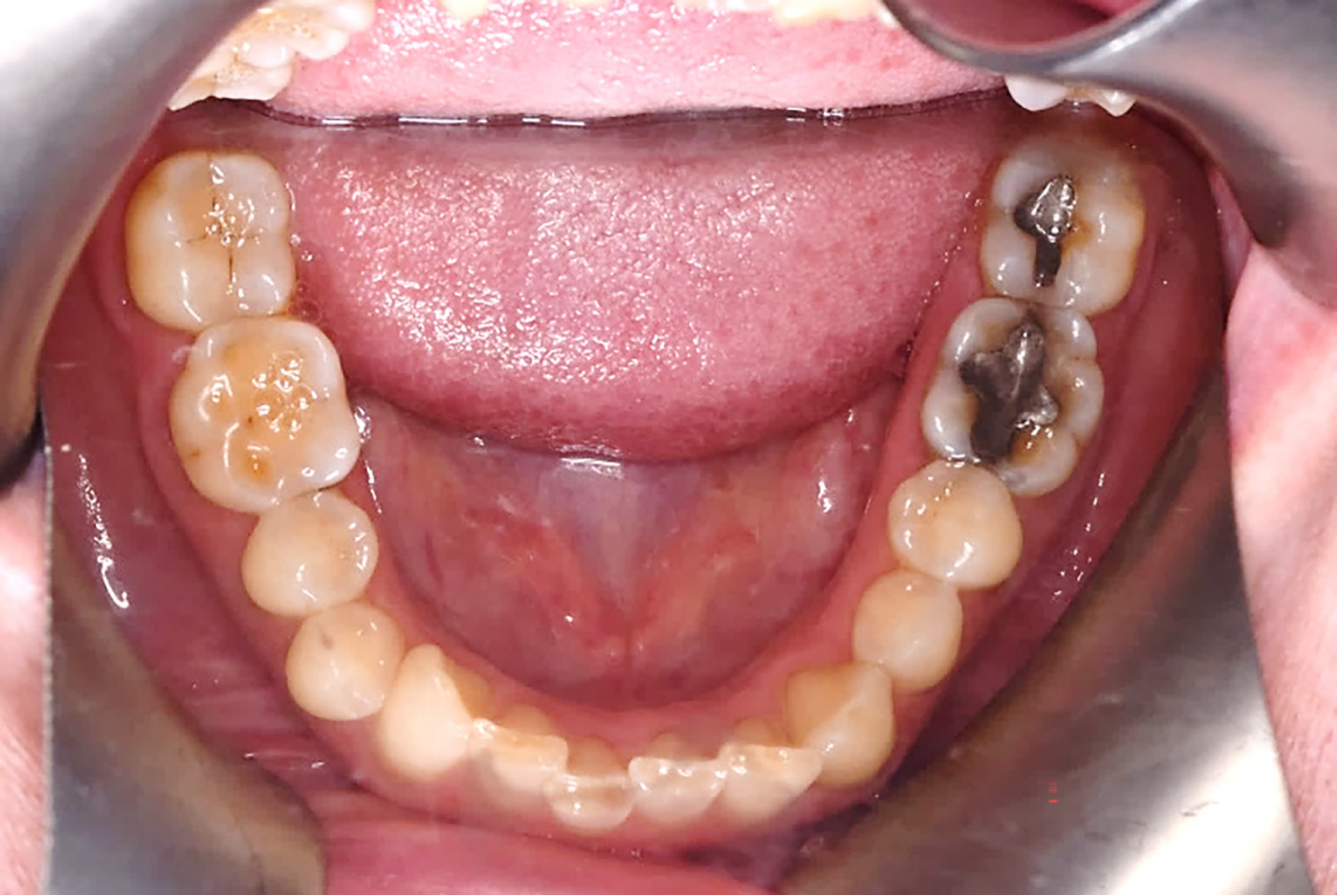
Mandibular occlusal view.

After examination, a list of patient problems was obtained. The patient had no endodontic or periodontal problems. Regarding the angle classification, the patient had a CL 2 DIV II jaw relationship along with occlusal plane cant, reduced overjet, severe deep bite, and anterior crowding (Figure 2).Attrition was seen at the incisal edge of anterior teeth and the occlusal surface of posterior teeth (Figure 3).

**FIGURE 4:**
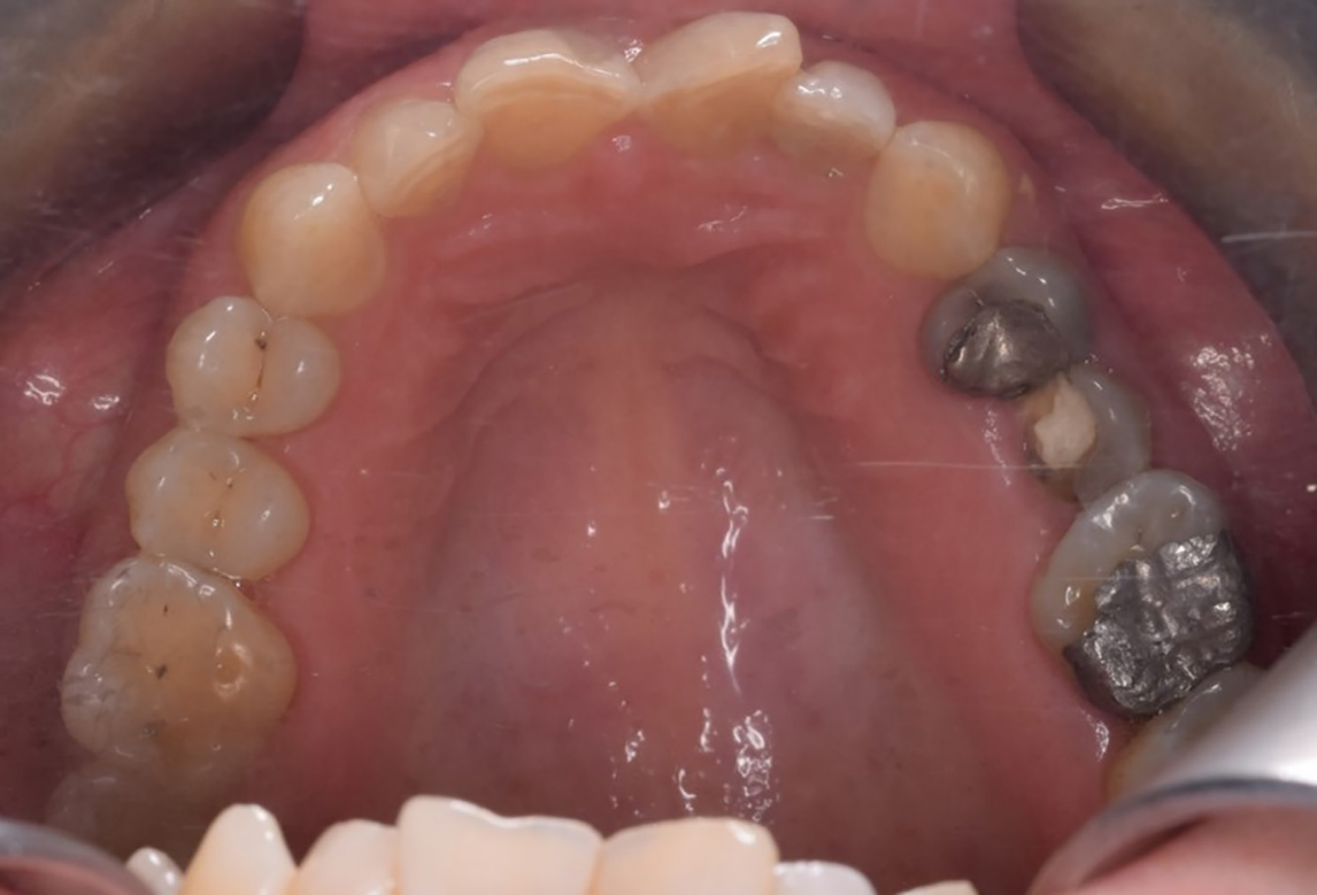
Maxillary occlusal view.

Likewise, abstraction was evident in the cervical region of posterior teeth, indicating that the patient had an occlusion problem. The fracture of the left upper second premolar was also observable (Figure 4).

The aesthetic characteristics of the patient were assessed from three macro‐, mini‐, and micro‐aesthetic views, and the following problems were reported for the patient:

1. *From a macro‐aesthetic view*: Asymmetrical eyes (the inter‐pupillary line was not parallel with the horizon plan).
2. *From a mini‐aesthetic view*: The incisal plane was not parallel with the horizon; the smile arch did not coincide with the lower lip; the presence of occlusal plane canting; more gingiva was shown at the right side; midline deviation; asymmetrical smile; the RED proportion was not respected.
3. *From a micro‐aesthetic view*: Discoloration of teeth; anatomical proportions were not respected; asymmetry in gingival height and zenith; the problems with crowding and the longitudinal axis of teeth.

### 2.1 The treatment plan selection

The examinations suggested that the lack of stable contacts in palatal segments of the upper anterior teeth caused the dental cingulum loss in the upper jaw dentition. This alteration led to the overgrowth of upper and lower teeth and ultimately aggravated the deep bite. During the examination of the casts, the CR contact of the lower anterior teeth on palatal tissues was observed.

**FIGURE 5:**
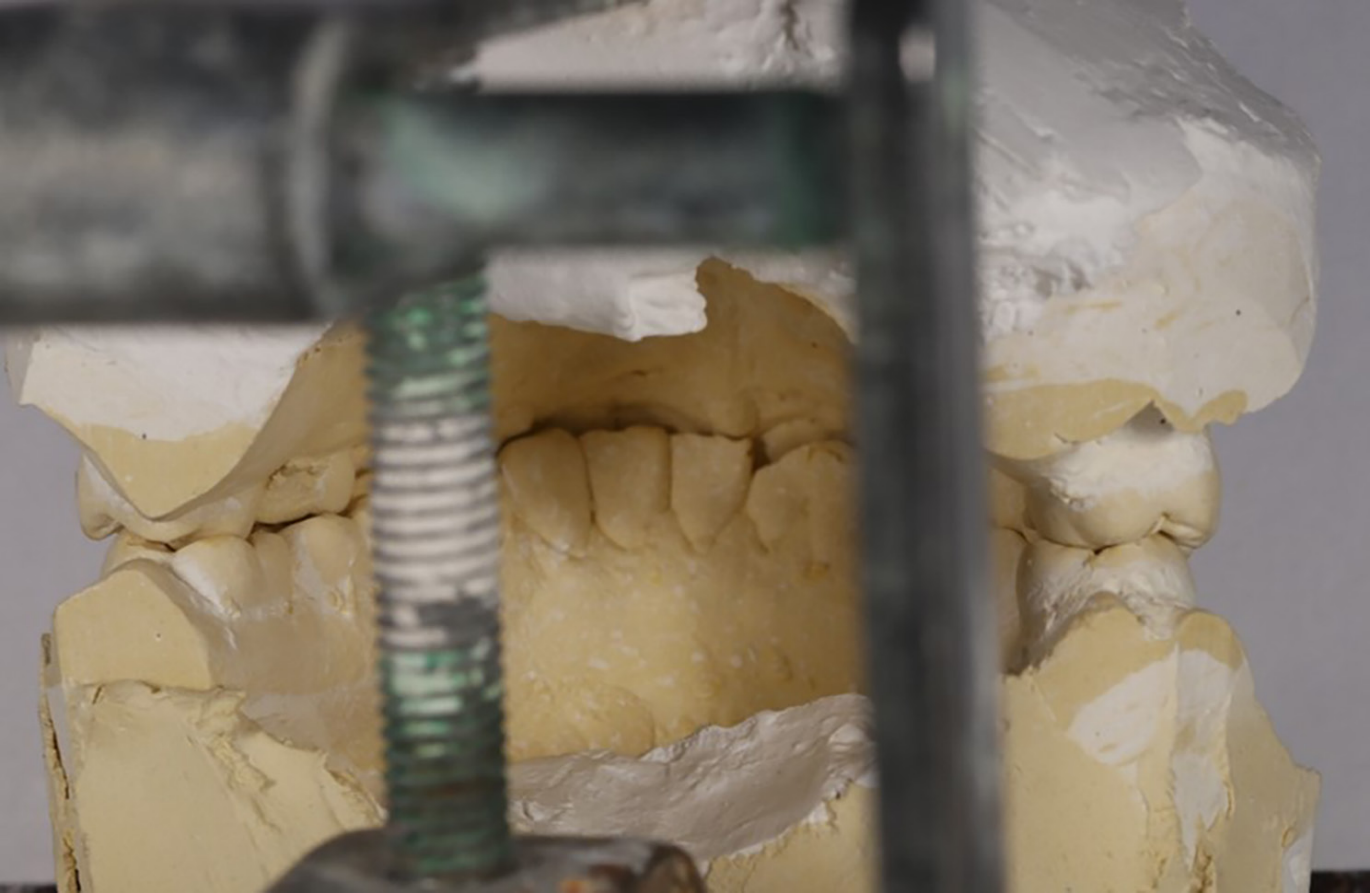
Diagnostic cast.

The aesthetic treatment plan will be preceded by creating a stable posterior and anterior occlusion. The first stage of treatment consisted of establishing stable contacts by cingulum restoration for upper anterior teeth while creating smooth edges for the lower anterior teeth to ensure stable contacts of lower anterior teeth on palatal surfaces of the upper anterior teeth. Thus, after the clinical examination of the patient and analysis of the study casts, the Dahl technique was decided to be used for restoring and establishing occlusal contacts in correct positions to modify the occlusion and open the bite (Figure 5).

To increase the bite in the original Dahl technique, a partial metal appliance is placed on the palatal surfaces of the upper anterior teeth to create an inter‐occlusal space of 1.8–4.7 mm. Thus, a combination of interior intrusion (40%) and posterior eruption (60%) will be achieved. In this technique, the average time of achieving an ideal occlusion is 6 months for most people, and the chief reason for treatment failure is the non‐cooperation of the patients in using the appliance which is why we altered the anterior guidance to act as a fixed anterior bite plan and to have stable anterior contacts.^13^ Alternatively, this technique allows for cingulum restoration, providing sufficient space for the posterior teeth eruption and anterior guidance correction by creating composite stops on the anterior teeth without the need to use an appliance. This, in turn, will increase the possibility of the patient's cooperation and improve the treatment effectiveness. The objective of this treatment was to provide the lost space due to tooth wear in the anterior, remove the posterior teeth from contact, and provide space for vertical movement (eruption) of teeth.

**FIGURE 6:**
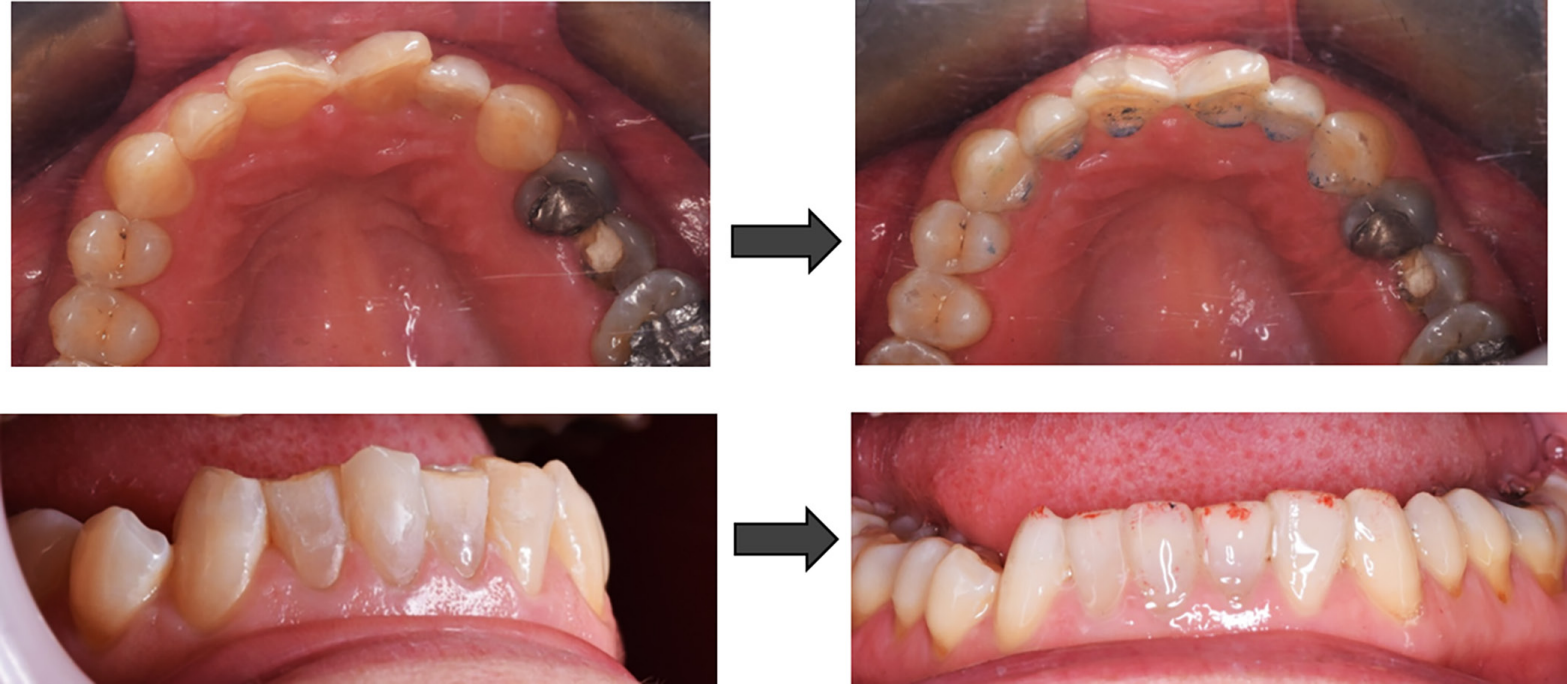
Occlusal adjustment. Creation of composite stops on anterior teeth: Resulting on enough space for posterior teeth eruption, restoring cingulum and anterior adjustment.

Both mandibular and maxillary anterior teeth were restored in this patient, and the composite stops were created on the anterior dentition of both jaws. The patient's bite was opened after this restoration, and by making enough space between the teeth in both jaws' posterior regions, it also became possible to restore the patient's worn‐down posterior teeth (Figure 6).

**FIGURE 7:**
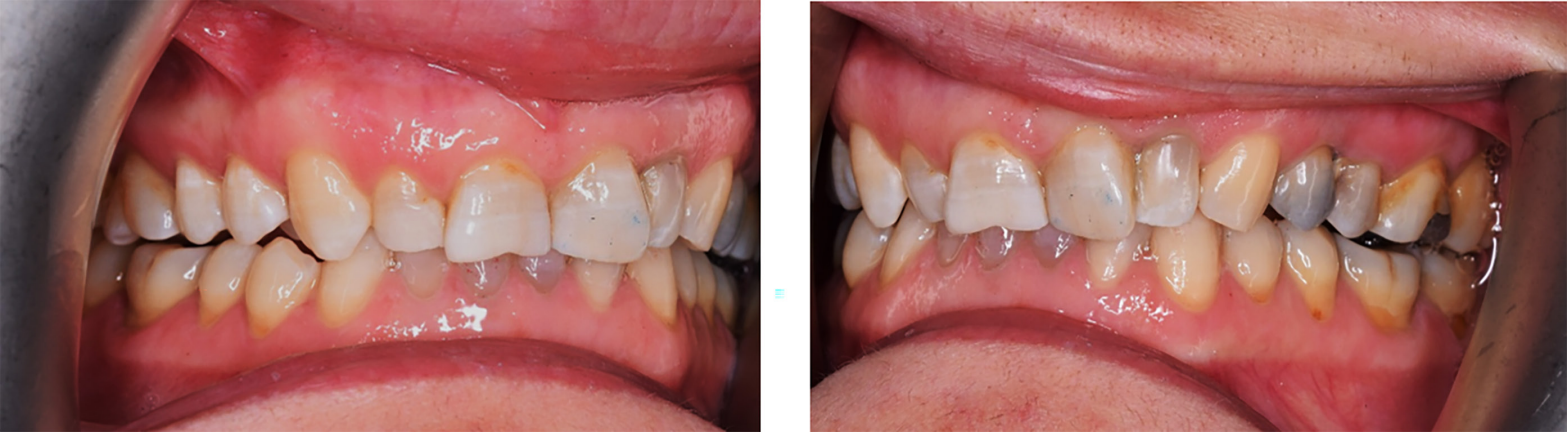
Immediately after tooth contact adjustment with composite.

**FIGURE 8:**
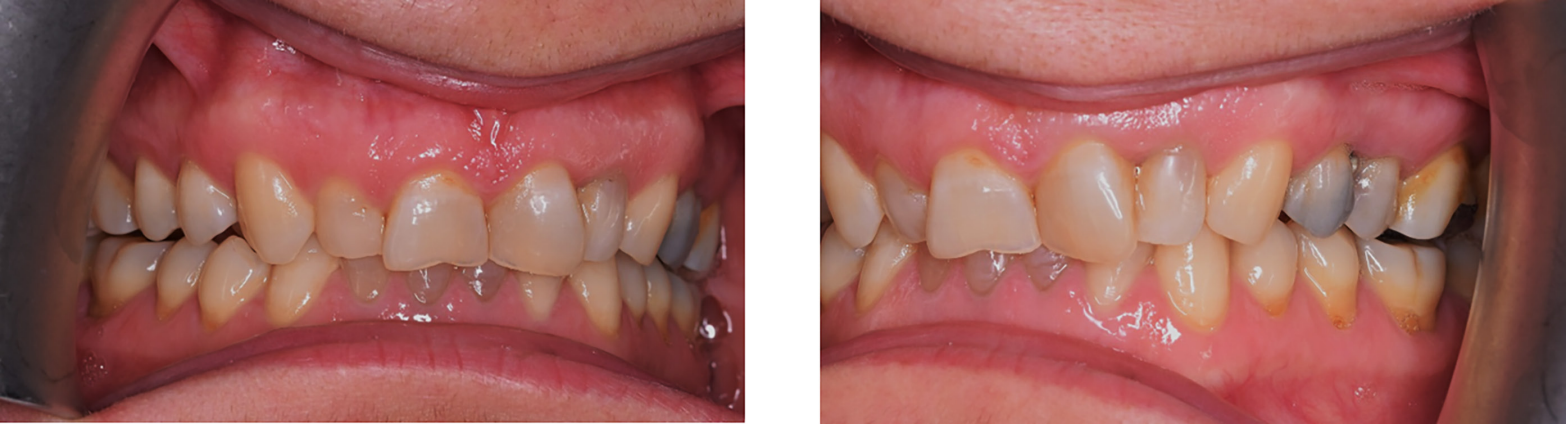
Four weeks after tooth contact adjustment with composite.

**FIGURE 9:**
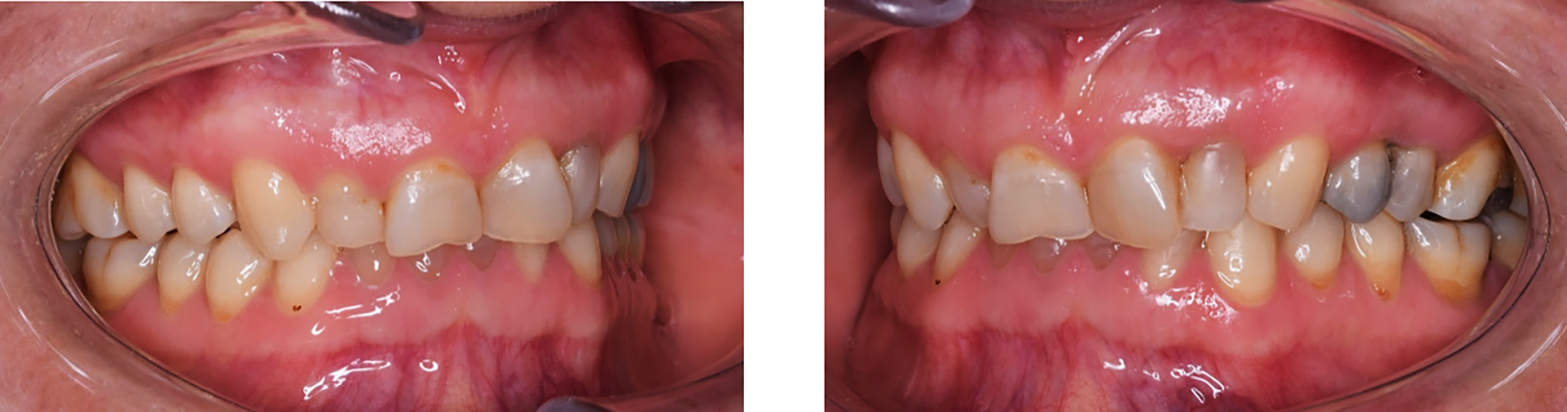
Ten weeks after tooth contact adjustment with composite.

**FIGURE 10:**
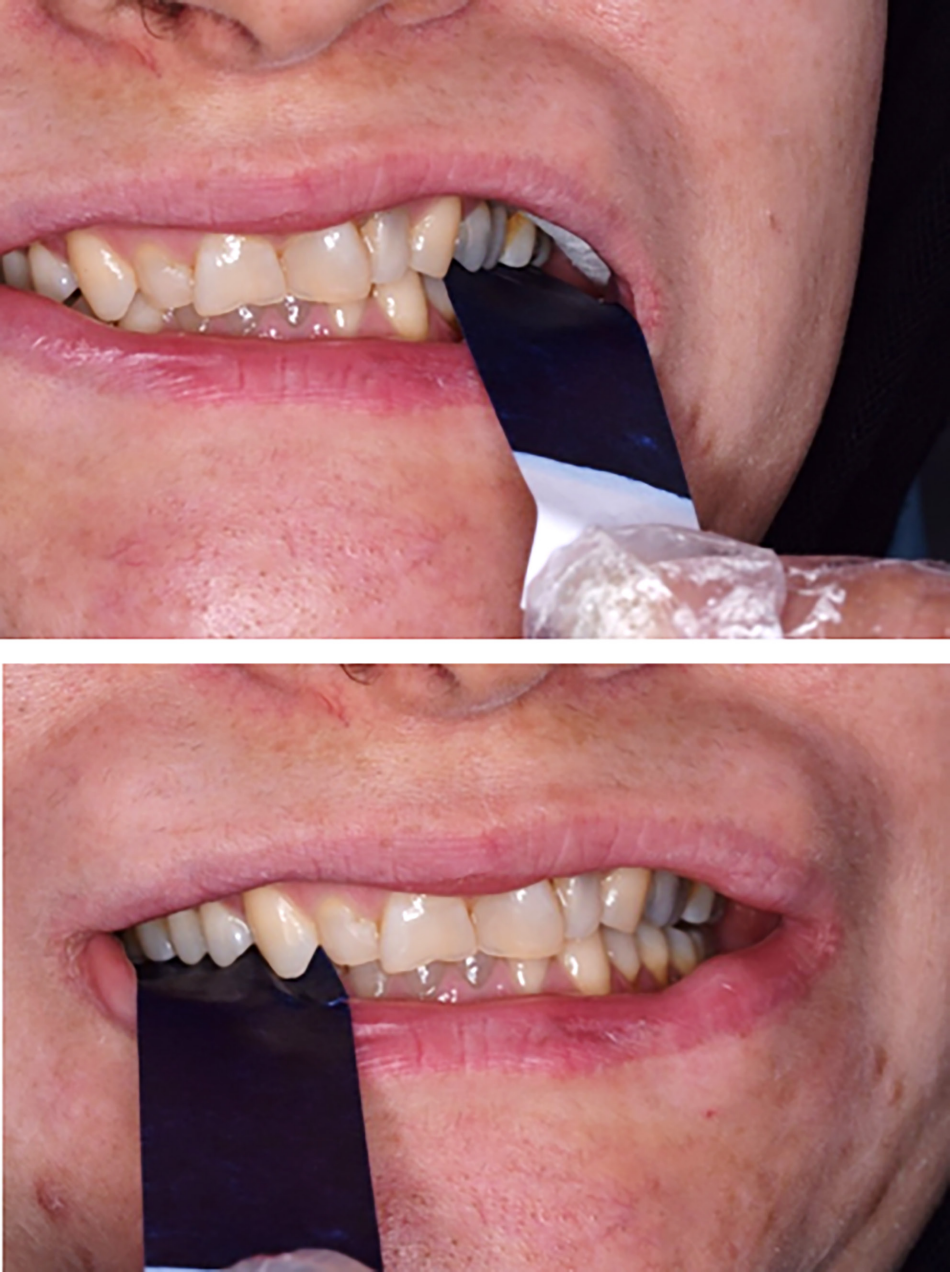
Reestablishment of occlusal contacts after 10 weeks.

**FIGURE 11:**
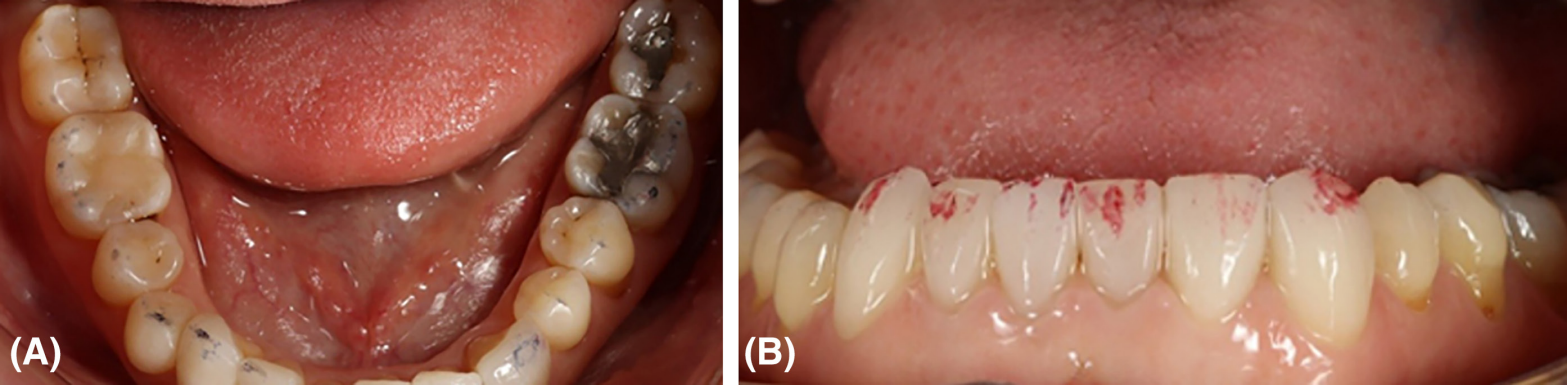
Occlusion after cementation. (A) Dots in the back. (B) Lines in the front.

**FIGURE 12:**
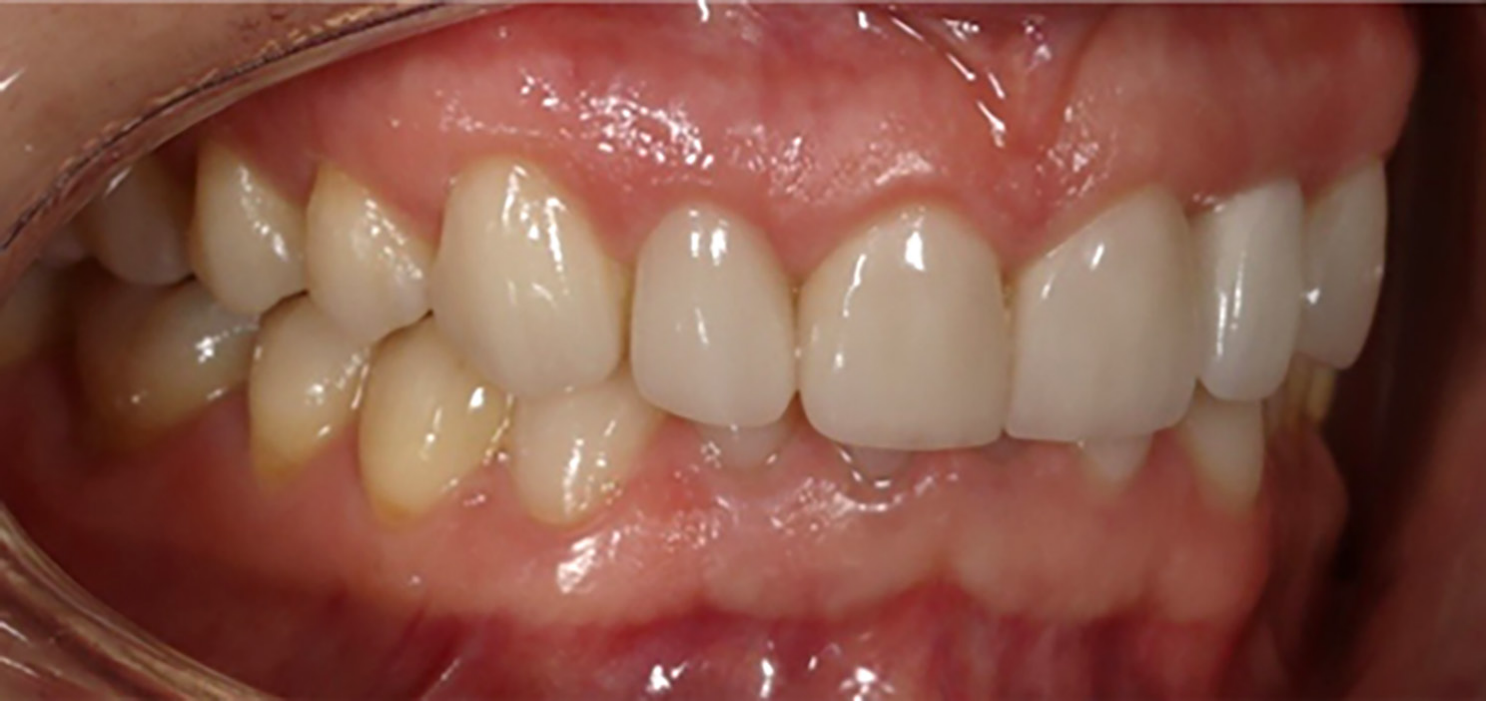
Final result.

A follow‐up was conducted on the patient to evaluate and compare the level of occlusal contacts. The observations were recorded by photography before, immediately after, at 4 and 10 weeks after performing composite corrections (Figures 7, 8, 9). Tooth contacts were found to be re‐established at the end of Week 10 (Figures 10 and 11). Then soft tissue CL was carried out by the electro‐surgery device to correct the gingival levels of the left central, lateral, and canine teeth of the patient. After shaping and preparing the anterior teeth of both jaws, the impressions for the construction of ceramic laminates were taken by a 1‐stage technique using polyvinyl siloxane (PVS). After delivering the lithium di‐silicate laminates, a night guard was fabricated for the upper jaw, and its necessity and method of use were explained to the patient. The final result was very pleasant for the patient (Figure 12).

## 3 DISCUSSION

The present case report is concentrated on the treatment plan for a patient with a pathologic deep bite, progressive wear, and anterior deep bite. The pathological deep bite has signs and symptoms (including traumatic palatal soft tissues, unstable occlusion, tooth wear, and aesthetic issues) that their treatment process may demand decisions like orthodontic treatment or reshaping or compromised restorations.^14^ One of the challenges in the treatment of deep bite patients is the limited available space for placement of the required restorations. These conditions become increasingly complicated when the occlusal wear occurs in a deep bite occlusion.

The compensatory dentoalveolar growth, which occurs continuously and following wear issues, fills the space required for the reconstruction of the worn teeth. Under these circumstances, the following treatment options can be used to provide the space needed for retention, stability, and aesthetics:

1. Orthodontic intrusion and proclamation of the supra‐erupted teeth.
2. Teeth reduction in the same or opposite arch, occlusal plane correction, and occlusal reconstruction with and without surgical CL.
3. Temporally increasing the VDO in posterior teeth (using Dahl appliance, direct or indirect restorations with full or partial coverage, and orthodontic extrusion of posterior teeth)
4. Elective endodontic procedure to provide adequate space and grip from inside of the dental structure using the post‐retained restorations or Richmond crown.^15^, ^16^

Each of these treatment options has its particular indications and considerations. On the other hand, the wear of buccal surfaces of the anterior teeth in the lower jaw (which also occurred in the present case) is another problem that demands reconstructing anterior guidance.

VDO can be increased using the fixed prosthesis or removable appliance. Concerning the method applied to increase the VDO, those studies that used the fixed prosthesis reported fewer complications compared to those that used the removability feature. The Dahl technique utilizes a local appliance or restoration in the supra‐occlusion position on the anterior teeth, allowing the posterior teeth to extrude and the anterior teeth to intrude to achieve full arch contacts over a while. However, the success of this appliance is fully dependent on the patient's cooperation. Several complications have been reported, including TMJ dysfunction, possible tenderness during treatment, sensitivity, unpredictable tooth movement, and tilt or drift of other teeth.^17^ Another method for increasing the VDO includes orthodontic extrusion, usually the posterior extrusion of teeth combined with anterior–posterior repositioning of anterior teeth with limited intrusion. Nevertheless, the intrusion is much more complicated in adults, and orthodontic treatment requires a time duration of 6–12 months, which is significant for equalizing the mesiodistal spacing that occurs during the teeth forward movement.^18^ A better therapeutic option to gain space in this patient is the fixed Dahl technique, which is an anterior bite block in order to create space in the anterior section allowing us to properly restore the patient's anterior guidance and aesthetics while posterior teeth are untouched and in contact.

Space gained by this technique in 40% anterior intrusion and 60% extrusion of posterior teeth in 10 week time period. By literature, during a year original VDO is regained by muscles. It is by far the most conservative while effective treatment plant for this case.

Conservative ceramic coverage is the most durable aesthetic option among the mentioned restorative methods. However, design selection, proper preparation, skillful ceramist, and choosing the ceramic material play a significant role in the long‐term success of this treatment. Numerous factors, including the amount of remaining tooth structure, restoration material type, finish line design, restoration thickness, and cement types, can affect the mechanical behavior of occlusal veneers. Various ceramic materials can potentially be used in low thickness for high‐stress situations. High‐glass ceramics have lower compressive strength, whereas the low‐glass types such as lithium disilicate (LDS), zirconia‐reinforced lithium silicate (ZLS), and hybrid ceramics offer appropriate aesthetics and much higher strength. The bonding of glass ceramics is one of their significant properties for partial coverage restoration applications.^19^

While composite resin, LDS, ZLS, and hybrid ceramics can be employed for posterior occlusal coverage, disagreement exists in the literature regarding the superiority of composite resin or ceramic restoration.^20^ In the present case, composite occlusal veneers were utilized to increase the VDO, which seems to be a conservative treatment. Given the higher aesthetic characteristics of ceramic laminates, disilicate lithium laminates were eventually constructed for the anterior teeth of both jaws. However, both approaches had a reliable performance for both functional and aesthetic purposes. Long‐term clinical trials are needed to find the best material, thickness, and treatment plan.

For adequate oral function and to avoid negative effects like implant overloading, proper implant occlusion is crucial.^21^ In addition, compared to externally attached implants, internally connected implants demonstrated decreased marginal bone loss.^22^

Also, this problem can solve with the use of digital dentistry. Patients' own aesthetic evaluation showed satisfactory results that were fully compatible with those reported in the literature. Specifically, the customer satisfaction analysis showed greater satisfaction, in terms of comfort/discomfort, with the digital protocol. Still, further long‐term prospective clinical studies, are needed to investigate the full potential of the digital procedure and to corroborate these preliminary findings.^23^

## 4 CONCLUSION

Dealing with attrition problems, the plate technique, if used correctly, can be an alternative solution for the treatment of occlusal and attrition problems, especially in mild and moderate cases of localized tooth wear. The clinical ease and the fact that composite restorations can be customized and modified intraoral over a week or two provide better controllability of the treatment outcome. This can be regarded as the ultimate preventive dentistry. So, this treatment plan while being conservative and providing satisfactory esthetic, helps to rebuild anterior guidance. The patient was excited about the final treatment outcome. In addition to the aesthetical aspect, this treatment plan was simple, conservative, cost‐effective, and durable and could prevent further anterior and posterior progressive teeth wear.

## AUTHOR CONTRIBUTIONS

**Mahla Rezaei:** Data curation; writing – review and editing. **Hossein Chalakinia:** Investigation; methodology; project administration; supervision. **Maedeh Doost Mohammadi:** Validation; writing – original draft. **Farzaneh Khosravi:** Investigation; writing – original draft.

## FUNDING INFORMATION

Except for the financial support provided for the treatment of the case patient by Mashhad University of Medical Sciences, no other financial support was provided from other institutions.

## CONFLICT OF INTEREST STATEMENT

The authors declare no conflicts of interest.

## CONSENT

Written informed consent was obtained from the patient to publish this report in accordance with the journal's patient consent policy. The patient/participant is unable to give written consent.

## Data Availability

All the data used in this study are available and upon reasonable request from the corresponding author, the data will be made available to the applicant.
